# Calibrating emergent phenomena in stock markets with agent based models

**DOI:** 10.1371/journal.pone.0193290

**Published:** 2018-03-02

**Authors:** Lucas Fievet, Didier Sornette

**Affiliations:** 1 Department of Management, Technology and Economics, ETH Zurich, Zurich, Switzerland; 2 Swiss Finance Institute, Geneva, Switzerland; Central South University, CHINA

## Abstract

Since the 2008 financial crisis, agent-based models (ABMs), which account for out-of-equilibrium dynamics, heterogeneous preferences, time horizons and strategies, have often been envisioned as the new frontier that could revolutionise and displace the more standard models and tools in economics. However, their adoption and generalisation is drastically hindered by the absence of general reliable operational calibration methods. Here, we start with a different calibration angle that qualifies an ABM for its ability to achieve abnormal trading performance with respect to the buy-and-hold strategy when fed with real financial data. Starting from the common definition of standard minority and majority agents with binary strategies, we prove their equivalence to optimal decision trees. This efficient representation allows us to exhaustively test all meaningful single agent models for their potential anomalous investment performance, which we apply to the NASDAQ Composite index over the last 20 years. We uncover large significant predictive power, with anomalous Sharpe ratio and directional accuracy, in particular during the dotcom bubble and crash and the 2008 financial crisis. A principal component analysis reveals transient convergence between the anomalous minority and majority models. A novel combination of the optimal single-agent models of both classes into a two-agents model leads to remarkable superior investment performance, especially during the periods of bubbles and crashes. Our design opens the field of ABMs to construct novel types of advanced warning systems of market crises, based on the emergent collective intelligence of ABMs built on carefully designed optimal decision trees that can be reversed engineered from real financial data.

## Introduction

Complementary to the influential dynamic stochastic general equilibrium modeling in macroeconomics, agent-based models (ABMs) are particularly suitable to account for bounded rationality and adaptiveness of interacting agents [1–3], as well as out-of-equilibrium phenomena. Let us mention in particular that ABMs could in theory forecast regime changes from first principles [4]. ABMs are particularly well-suited to describe bubbles and crashes and to analyse the role of herding among some market participants and the influence of arbitrageurs in the presence of noise traders [5–8]. ABMs also provide a general framework to analyse the mechanisms at the origin of the stylized facts observed in stock markets, such as volatility clustering and fat tails of the distribution of returns.

Building on minority and majority ABMs derived from the El Farol Bar problem [9], a number of promising results have been obtained for this class of ABMs: (i) mixed game models with minority and majority agents exhibit trends and bubbles [10, 11]; (ii) majority agents with a delayed action have been found to be crucial for the existence of transitions between herding and contrarian regimes [12]; (iii) significant out-of-sample predictive power on hourly Forex data seems to be present [13]; (iv) predictability has been found to increase prior to large changes [14]; (v) pockets of predictability have been documented when the agents’ actions decouple from past returns [15]; and (vi) significant directional accuracy can be obtained with a single agent model [16, 17].

Other progresses involve attempts to solve the vexing issue of ABM calibration to empirical data [18]. One strategy is to link certain ABMs, using aggregation and linearization, to stochastic processes that can themselves be calibrated using standard statistical methods [19]. The advantage of deriving a linear model from an ABM is that the parameters are interpretable in terms of the relative influence of fundamentalist and noise traders [20]. While the resulting calibration supports the hypothesis of the existence of strong social interaction in short-run sentiment [21], the model is limited as it does not over-perform the random walk null in out-of-sample prediction benchmarks. Recent efforts using the generalized method of moments could however achieve volatility forecasting performance similar to that obtained with the standard GARCH model, while providing additional information on the trend following component [22].

Recent research has explored multiple paths to perform a true non-linear calibration of the BH (Brock and Hommes) agent based model to empirical data. One approach is to use gradient descent types of algorithm to minimize a constrained non-linear cost function around the model equilibrium [23, 25]. The in-sample calibration results indicate a predominance of trend following and a high risk aversion [25]. The out-of-sample validation shows a significant reduction in the relative error with respect to a random walk forecast [23]. A second, computationally more intensive approach consists in using a general non-parametric simulated maximum likelihood to recover the model parameters even when no closed form solution of the objective function exists [26]. The simulations show a good performance at recovering the parameters. A third approach uses Bayesian estimation techniques, selecting adequate prior parameter distributions in a simulated context before applying the estimation to real financial data [27].

Despite the recent progresses, the use of ABMs in economics and finance, in particular to inform policy decisions, remains scarce if not entirely absent, due to the above mentioned difficulties in calibrating them to empirical data [18] and the existence of local instabilities and of chaotic global dynamics, even in minimal ABMs with adaptive belief systems [28, 29]. A further difficulty is the comparison of structurally different models. This problem has recently been addressed with the introduction of a Markov information criterion that applies to the model outputs alone [24].

Here, we provide an important advance towards normalising the use of minority/majority type of ABMs by developing a bottom-up methodology that validates the usefulness of such ABM representations of financial markets. Our first innovation is to exhibit an explicit mapping of agents onto optimal decision trees, which allows us to achieve an efficient calibration of single agent models as a first step. This representation makes possible a systematical exploration of all single agent models to identify the statistically significant deviations from efficient markets, within a rigorous framework including corrections for multiple testing. The second innovation stems from the use of risk adjusted out-of-sample trading performance as a criterion to detect truly predictive models. This approach allows us to compare structurally different models, similarly to the Markov information criterion [24].

These two steps prepare the ground for constructing and exploring two-agent models with heterogenous investment horizons, decision processes (games) and decision lags. The heterogeneity in investment horizon is known in particular to be crucial in ABMs to reproduce long-term memory observed in the stock market [19]. The resulting multi-agent mixed game ABMs exhibit remarkable predictability skills arising from the collective intelligence of two-agents groups that transcend all one-agent strategies, allowing the detection of extreme regime changes, such as market crashes. Our results show the existence of two-agent models with significant superior predictive power over all single agent models, however do not yet imply the possibity of calibrating such models ex-ante. The calibration of such two-agent models is a promising path to explore, and will certainly benefit from a Bayesian framework [27].

## Representing agents as decision trees

In minority [30–32] and majority [12, 33] agent models, the global information shared by all agents is the history ${\vec{r}}_{t}=\left\{r_{t-\varrho +1},\ldots ,r_{t}\right\}$ of the most recent *ϱ* outcomes (where *ϱ* is the memory length), given as a binary sequence ${\vec{r}}_{t}\in {\left\{-,+\right\}}^{\times \varrho }$ of up and down moves. An individual strategy associates to each history an action to buy or sell. An agent possesses a private set of strategies, and keeps track of their performance on the past window of size *L*, using a payoff function *π*, which depends on the game played by the agent. At each time step, an agent buys or sells stock following his best strategy. Liquidity is often modeled using a trading threshold below which an agent would not trade [13], however this feature is unnecessary for the present work.

### Strategy space

**Fig 1:**
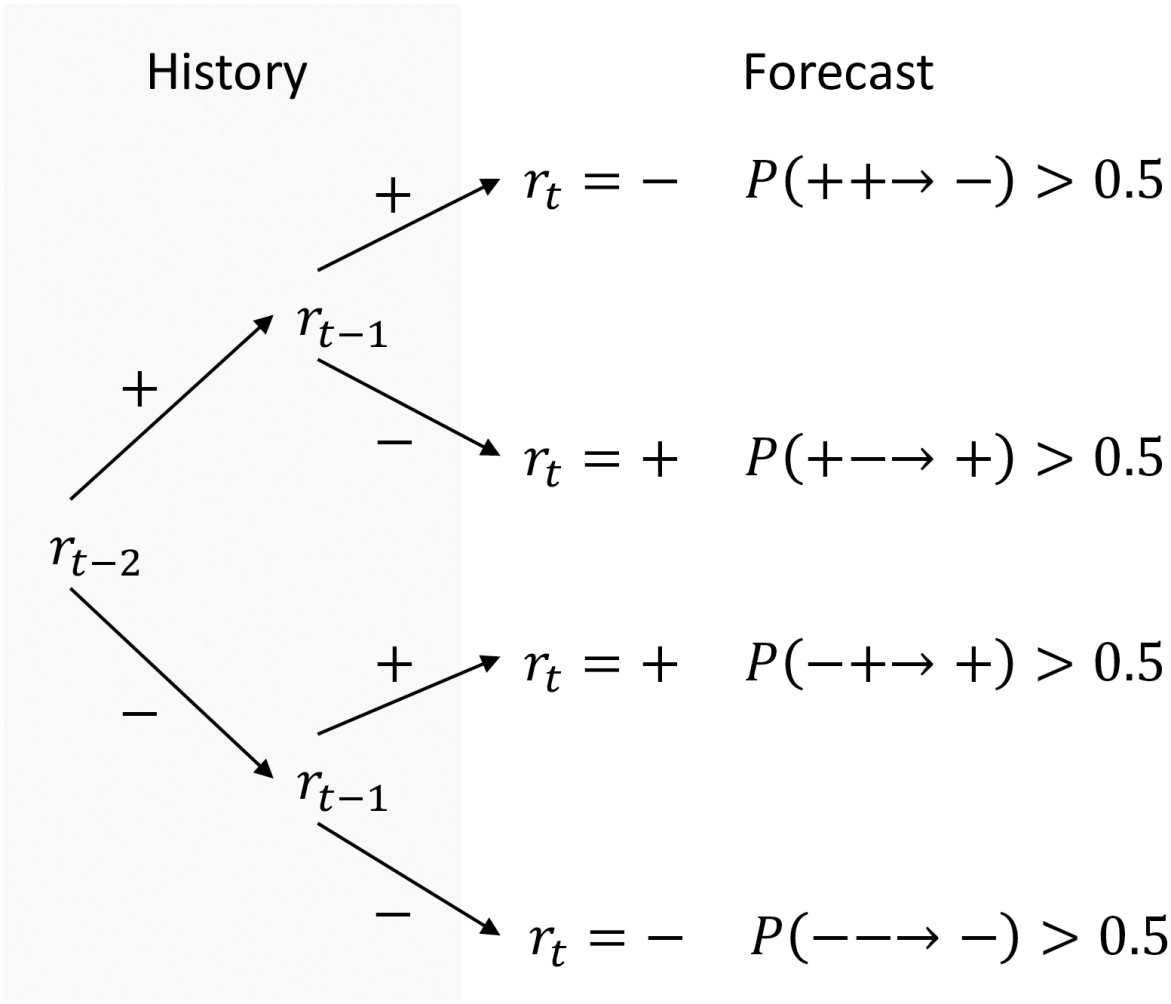
Arbitrary strategy with two lags *ϱ* = 2 represented as a decision tree. Each of the 2^2^ = 4 possible histories (*r*_*t*−1_, *r*_*t*_) is assigned to a buy or sell action determined by the class (up or down move) with the highest probability. The class probabilities are computed from the calibration data using Eq (8), and in the present example are assumed to fullfil the shown inequalities.

For a fixed number *ϱ* of lags, a strategy is a function

$$\begin{array}{c} s:{\left\{-,+\right\}}^{\times \varrho }\to \left\{-,+\right\}, \end{array}$$ (1)

associating an up or down prediction to each history. An example of a two lag strategy is presented in Fig 1.

The resulting strategy space

$$\begin{array}{c} S^{\varrho }:=\{s|\forall s:\;{\left\{-,+\right\}}^{\times \varrho }\to \left\{-,+\right\}\} \end{array}$$ (2)

contains $\left|S^{\varrho }\right|=2^{2^{\varrho }}$ elements. In typical multi-agent simulations, some heterogeneity among agents is introduced by assigning to each agent *A*_*i*_ a subset $S_{i}^{\varrho }\subseteq S^{\varrho }$ of all possible strategies.

### Minority & majority payoff

Heterogeneity of beliefs among market participants is modeled by two opposite games: the minority game, where agents believe that recent trends reverse (anti-persistency); and the majority game, where agents believe that recent trends continue (persistency). The games are defined by a payoff function *π*: {−, +}^×2^ → {−, +} that associates to a true return *r*_*t*_ and predicted return ${\tilde{r}}_{t}$ the unitary gain or loss $\pi (r_{t},\;{\tilde{r}}_{t})$. The minority payoff function (MIN) is defined as

$$\begin{array}{c} \pi_{\text{MIN}}(r,\;\tilde{r})≔1-2\delta_{r,\;\tilde{r}}, \end{array}$$ (3)

and the majority payoff function (MAJ) as

$$\begin{array}{c} \pi_{\text{MAJ}}(r,\;\tilde{r})≔-\pi_{\text{MIN}}\left(r,\;\tilde{r}\right)=2\delta_{r,\;\tilde{r}}-1\;, \end{array}$$ (4)

where *δ* is the Kronecker delta. By construction, the actions of a minority agent are obtained by reversing all the actions of the equivalent majority agent.

### Delay

Agents with delayed actions are a crucial ingredient in mixed game models to observe speculative up-trends followed by a correction [12]. A delayed strategy *s* forecasts the direction of the return *d* steps ahead as ${\tilde{r}}_{t+d+1}=s\left({\vec{r}}_{t}\right)$, where *d* is the delay parameter. This is a simple way to represent the lag between analysis, decision and action that is often imposed by operational constraints in real-world strategy implementations.

### Strategy performance & selection

The agents rank their strategies by past performance and trade one step ahead using the best strategy. The performance *U* of a strategy *s* at time *t*, for the payoff function *π*, with a delay *d*, on the past window of size *L*, is given by

$$U_{\pi ,\;d,\;L}\left(s,\;t\right)=\frac{1}{\tilde{L}-d}\overset{t-1-d}{\underset{j=t-1-\tilde{L}}{\sum }}\pi (r_{j+1+d},\;s({\vec{r}}_{j}{}_{,\varrho })),$$ (5)

where ${\vec{r}}_{j,\;\varrho }=\left(r_{j+1-\varrho },\ldots ,r_{j}\right)$, and $\tilde{L}=L-\varrho$. Returns before the past window of size *L* are considered as obsolete for current events. At each time step *t*, the prediction ${\tilde{r}}_{t+1}^{i}$ of agent *i* is given by

$$\begin{array}{c} {\tilde{r}}_{t+1}^{i}=s_{i}^{*}({\vec{r}}_{t-d,\;\varrho }),\;\text{where}\;s_{i}^{*}=\underset{s^{\prime }\in S_{i}^{\varrho }}{\text{argmax}}\;U_{\pi ,\;d,\;L}\left(s^{\prime },\;t\right), \end{array}$$ (6)

which is the prediction of the agent’s strategy with the highest performance on the past window of size *L*.

### Agent as optimal decision tree

When calibrating a model with a single agent, heterogeneity in the agent’s strategies is not required, and the agent can be endowed with the knowledge of all strategies. Models with a partial strategy set are possible, but not optimal from a decision theory point of view. Moreover, there are 2^*ϱ*^ partial strategy sets for a lag *ϱ*, which would drastically increase the number of models to analyze (which is nonetheless feasible). Hence, the present work focuses on analyzing single agent models with the full strategy space.

The fact that a strategy is equivalent to a decision tree is illustrated in Fig 1, where each branch corresponds to a single history, and the terminal node of a branch assigns the action following its history. As proven in (S1 Appendix), the best performing strategy in $S^{\varrho }$ for the majority payoff is always the optimal fixed classification tree T(·), as constructed with the Classification And Regression Tree algorithm (CART, see [34]) applied to the binary returns in the past window of size *L*. The samples used for training the decision tree at time *t* are given by

$$\begin{array}{c} X_{t}=\{({\vec{r}}_{t-\tilde{L},\;\varrho },\;r_{t-\tilde{L}+d+1}),\;\ldots ,\;({\vec{r}}_{t-1-d,\;\varrho },\;r_{t})\}. \end{array}$$ (7)

For a given training sample **X**, the CART algorithm computes the class probabilities for {−, +} in each branch (i.e. history). The class probabilities for a history $\vec{r}$ are determined by the ratio

$$\begin{array}{c} P\left(\vec{r}\to -\right)=1-P\left(\vec{r}\to +\right)=\frac{\left|\left\{X\in X|X=(\vec{r},-)\right\}\right|}{\left|\{X\in X|X=(\vec{r},\cdot )\}\right|}, \end{array}$$ (8)

where |{…}| denotes the cardinal of the set {…}. Hence, a decision tree with binary classes is determined by a total of 2^*ϱ*^ probabilities, one for each branch. An optimal majority tree predicts for each branch the class with the highest probability, while the optimal minority tree predicts the class with the lowest probability. Ties are settled by a random draw.

A detailed treatment of autoregressive decision trees and the CART algorithm is provided in [35], in which it is shown that decision trees are robust predictors of time series with linear autoregressive correlations as well as for time series with non-linear sign correlations, the later being often undetectable by linear autoregressive models. Hence, decision trees constitute a solid foundation for building predictive strategies for agents in ABMs.

Further on, the description of a minority/majority agent as a decision tree provides a natural starting point to integrate such agent based models to the field of machine learning. The multi-agent models are closely related to machine learning methods such as bagging or boosting. Nonetheless, important differences remain, as typical calibration procedures of multi-agent models calibrate all agents synchronously and not independently, or successively, as is the case for bagging, respectively boosting.

## Performance analysis

### Trading strategies

The performance of a single agent model is evaluated by trading based on the one step ahead forecasts using a rolling window of size *L* for calibration, and taking a long or short position defined by $s_{t+1}=\text{sign}({\vec{r}}_{t+1})$. This produces a sequence of binary trading signals ${\left\{s_{t}\right\}}_{L+1}^{T}\in {\left\{-,+\right\}}^{T-L}$ that define the corresponding trading strategy on the market returns ${\left\{r_{t}\right\}}_{L+1}^{T}$. Hence, a strategy is defined by a payoff function (MIN or MAJ), the number of lags *ϱ*, the calibration window length *L*, and the delay *d*. For further convenience, it is implicitly assumed that *L* initial returns have been cropped before the first forecast.

### Anomalous predictability

Detecting anomalies across a large number of experiments is challenging, as the probability of spurious anomalies increases with the number of tests. We use the step-down methodology to correct for multiple testing [36–38], which rejects as many null hypotheses as possible, at given significance level, without violating the familywise error rate.

We measure risk adjusted returns with the Sharpe ratio [39], and benchmark against the buy-and-hold strategy using a stationary block bootstrap of returns, rejecting at a significance level of 0.05. The bootstrap p-values express the probability of the strategies under-performing the buy-and-hold strategy, when sampling with replacement blocks of returns from the time period of interest. However, during market draw-downs, a large number of lucky strategies are short in the market, hence have significant Sharpe ratio, but without possessing any true skill in predicting daily returns. To further restrict the definition of an anomaly, besides abnormal Sharpe ratio, we require the simultaneous presence of anomalous daily directional accuracy at a significance level of 0.05 (S2 Appendix). Hence, a model is anomalous when its Sharpe ratio is statistically significant, and explained by its ability to predict the daily market return direction.

We search for anomalous periods using a rolling window with an initial size of one year (250 trading days), moving in steps of 10 trading days. In each window, the model returns are tested for the simultaneous occurrence of statistically significant Sharpe ratio and directional accuracy. For anomalous one year windows, the beginning is incrementally cropped, and the end incrementally cropped or extended, in steps of 10 days, until the window maximizes the test statistic. This procedure avoids introducing the test window size as an additional parameter. To ensure finding the window with the maximal test statistic, a brute force search is performed for windows up to three years. Window sizes much shorter than one year never produce significant results due to the small sample size, and time spans longer than three years less relevant for the understanding of bubble dynamics.

We remark that the bootstrap algorithm maintains the correlation structure between all strategies, and therefore the multiple testing adjusted p-values are not impacted by the choice of Δ*L*.

### Principal component analysis

The space of single agent models contains subsets of models with highly correlated forecasts. To disentangle the multiple overlaid dynamics, and extract the dominant components, we use principal component analysis (PCA, see [34, 40]). Denoting by $r^{i}=\left\{r_{1}^{i},\;\ldots ,\;r_{\tau }^{i}\right\}$ the returns of a strategy *s*^*i*^ over *τ* time steps in a set *S* of *N* strategies, the *M* principal components (PCs) are obtained by the minimization problem

$$\begin{array}{c} \underset{V_{M}}{\text{min}}\sum_{i=1}^{N}{\left\|\left(r^{i}-\bar{r}\right)-V_{M}V_{M}^{T}\left(r^{i}-\bar{r}\right)\right\|}^{2}, \end{array}$$ (9)

which finds the projection $V_{M}\in R^{M\times \tau }$, onto a subspace of dimensionality *M* that minimizes the reconstruction error. The projection *V*_*M*_ can be computed from the singular value decomposition of the matrix of observations [34]. To remove the overall bias common to all strategies, and extract the true principal components differentiating the strategies, the returns are centered with respect to the mean return $\bar{r}$ of all strategies.

The PCs capture time periods, potentially disjoint, with the highest correlation among a subset of strategies. Taking the mean return of the subset of strategies during the correlated periods, and zero outside the periods, is a PC that minimizes (9). The PCs allow us to determine the time periods with strongest inter-strategy correlation, and the projection coefficients determine the involved strategies for each PC.

## Two agent model

### Motivation

Many different traders populate real financial markets, each with their individual investment style. While investment styles certainly exist on a continuous spectrum, traders are commonly categorized into fundamentalists or trend-followers. Fundamentalists pursue long-term value based investments, while trend-followers pursue short term profits arising from price patterns, such as momentum, cycles, and possibly bubbles and crashes. Hence, let us consider a market in which the sign of the next returns is determined by two components as

$$\begin{array}{c} P(\vec{r}\to +)=P_{1}(\vec{r}\to +,\;L_{1},\;t)+P_{2}(\vec{r}\to +,\;L_{2},\;t), \end{array}$$ (10)

where *L*_1_ and *L*_2_ are time scale parameters, and each component acts at a different time scale (*L*_1_ < *L*_2_). When predicting such a time series with a single agent, three scenarios occur depending on the calibration window length *L*: *L* < *L*_1_ where the agent predicts well the dynamic of *P*_1_ but misses the dynamics of *P*_2_; *L*_1_ < *L* < *L*_2_ where the agent predicts the dynamics of *P*_2_ but misses the dynamics of *P*_1_; and *L* > *L*_2_ where the agent misses both dynamics. To optimally predict the dual overlaid dynamics, two agents at different time scales are needed. Similar results follow for two dynamics acting at identical time scale but different lags.

### Optimal weighting mechanism

For two individual agents, the optimal combination of their class probabilities (as defined by Eq (8)) is not given by their mean, because the distinct calibration lengths imply different accuracy on the estimated probabilities. The expected accuracy on the estimated class probabilities is determined by the average number of samples per history, which is given by $\frac{L}{2^{\varrho }}$. Hence, the class probability $P_{2A}^{-}$ of a down move in a two-agents model, weighting by accuracy the individual class probabilities of the two agents, is given by

$$\begin{array}{c} P_{2A}^{-}=\frac{2^{\varrho_{2}}\cdot L_{1}\cdot P_{A_{1}}^{-}+2^{\varrho_{1}}L_{2}\cdot P_{A_{2}}^{-}}{2^{\varrho_{2}}\cdot L_{1}+2^{\varrho_{1}}\cdot L_{2}}, \end{array}$$ (11)

where $P_{A_{1}}^{-}$ is the class probability of agent one, and $P_{A_{2}}^{-}$ of agent two. This weighting ensures that the class probabilities of the two single agents are weighted by their respective accuracy (approximately inverse to the square root of their confidence interval).

### Simulation study

To illustrate the superiority of the two agent model in a simulated context, we consider the return sign sequence defined by the periodic dynamics

$$\begin{array}{c} P(\vec{r}\to +)=\frac{1}{2}+\frac{1}{10}\cdot \sin(\frac{2\pi }{L_{1}t})+\frac{1}{10}\cdot \sin(\frac{2\pi }{L_{2}}t+\frac{\pi }{2}). \end{array}$$ (12)

We predict this time series for 2⋅*L*_1_ = *L*_2_ = 360 over 2500 days with a single agent model and a two agent model. The forecast performance is measured using a directional accuracy test as described in the S2 Appendix, averaged over 500 runs. Scaning all one agent models T(L∈{5,10,...,100}) of lag one (ϱ=1), and all two agent models $T^{\times 2}\left(\left\{L_{1},L_{2}\right\}\in {\left\{5,10,\ldots ,100\right\}}^{\times 2}\right)$ of lag one with mean weighting and optimal weighting, the following local maxima of directional accuracy are found

- p-value ≈ 0.15: T(L=40),
- p-value ≈ 0.22: T(L=70),
- p-value ≈ 0.16: $T_{mean}^{\times 2}(L_{1}=40,\;L_{2}=65)$,
- p-value ≈ 0.10: $T_{optimal}^{\times 2}(L_{1}=40,\;L_{2}=65)$,

The simulation shows that the optimally weighted two agent model outperforms (i.e. has the smallest p-value) the single agent models and the equally weighted two agent model. While real financial markets are more complex, this simulation highlights the advantage of a two-agent models to extract predictability from two overlaid dynamics.

## Empirical results

### Experiments

We search for anomalous predictability by analyzing the trading performance of all meaningful single agent models on the NASDAQ Composite index during the 20 year time period running from Jan. 1, 1995 to Dec. 31, 2015. The daily returns are obtained from Thomson-Reuters Eikon with dividend adjustment. A total of 2000 experiments is run, determined by the two games (MIN and MAJ) and the following parameter space.

At lag *ϱ*, the class probabilities for the 2^*ϱ*^ unique histories need to be computed using (8). The accuracy of the computed class probabilities depends on the average number of samples per leaf given by $\frac{L}{2^{\varrho }}$. In [35], the best decision tree models on the S&P 500 are found at *ϱ* ∈ {1, 2, 3} and *L* ≤ 500, significantly above an average of 20 samples per leaf. The upper bound of two years on the investment horizon is independently found optimal to reproduce stylized facts [19]. Hence, a calibration length of up to two years, and a maximum of four lags, constitute a reasonable search space that encompasses all potentially interesting models. Two strategies that only differ by their calibration window lengths *L*_1_ and *L*_2_ are highly correlated when |*L*_2_ − *L*_1_| < 10. Therefore, we use a step size of Δ*L* = 10, which provides a sufficient sampling of strategies, while avoiding to compare almost identical strategies.

For the delay parameter *d*, we test *d* ∈ {0, 1, 2, 3, 4}. Experiments in behavioral finance show that traders are slow to update their beliefs, a phenomenon known as conservatism [41]. It takes between two to five contrarian observations before a subject changes his opinion, which motivates the range of chosen delays.

### Single agent results

**Fig 2:**
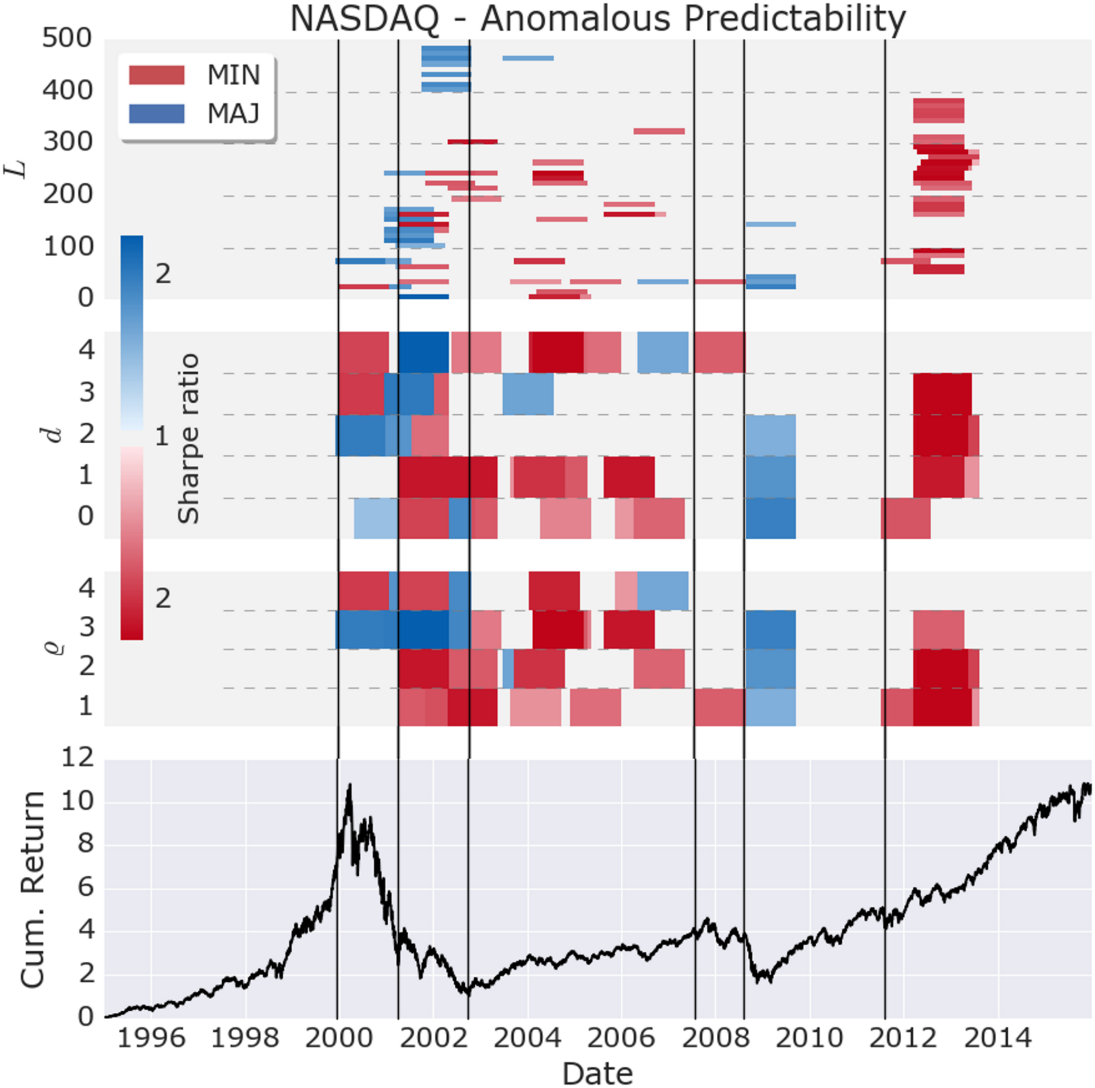
Anomalous time periods found in the NASDAQ (lower graph) with single agent models during the time period Jan. 1995 to Dec. 2015. A total of 2000 models are tested, resulting fromml: 2 games (MIN/MAJ) ×4 lags (*ϱ*) ×5 delays (*d*) ×50 calibration lengths (*L*). Using a rolling window of one year, 105 periods are found that exhibit statistically significant Sharpe ratio and directional accuracy, after adjusting for multiple testing. The intensity of the color is determined by the maximum Sharpe ratio of the out-performing models. The black vertical lines are given as a visual aid for the timing of market anomalies.

The experiments revealed a number of anomalous periods in the NASDAQ as shown in Fig 2. During up trending markets, instances of models with sufficient directional accuracy to beat the buy-and-hold market are rare, and may be spurious. However, during flat but volatile markets, and market crashes, strongly anomalous periods are found for both minority and majority games, robust with respect to large changes in the number of lags *ϱ*, delays *d*, and calibration lengths *L*.

Anomalous market dynamics are found precursory to the dotcom bubble and the financial crisis, providing empirical support that large changes in the stock market are preceded by increased predictability as theorized previously [14]. The events are preceded by an anomalous delayed market anti-persistency (minority game) at a short time scale of 30 to 40 days. We remark that the term minority game can be misleading, as an anomalous minority signal implies that a dominant fraction of agents believes in a market reversal during a significant number of days. A more appropriate name would be contrarian game, or reversal game. The delayed minority anomaly signals that a sizable fraction of market participants start to be contrarians. This dynamic breaks the growth phase of the bubbles, and transitions the market to a flat regime with high volatility. The contrarian anomalies are followed by an anomalous majority signal at shorter delay and longer time scale. The majority anomaly can be interpreted as a majority of traders flipping to the contrarian side, exiting the market, and hence bursting the bubble.

As a cross-check, the same search for anomalous models was performed on the S&P 500 and Dow Jones. The findings remain robust across all three equity indices, as shown in S1 Fig, respectively S2 Fig.

### Principal component analysis

The dynamics during the dotcom bubble and financial crisis are characterized by the simultaneous occurrence of the anomalous performance of multiple single agent models, and the transition over time between anomalies at different time scales, lags, and delays. A better understanding of the interplay between the different anomalous regimes is crucial for the construction of multi-agent models performed below.

**Fig 3:**
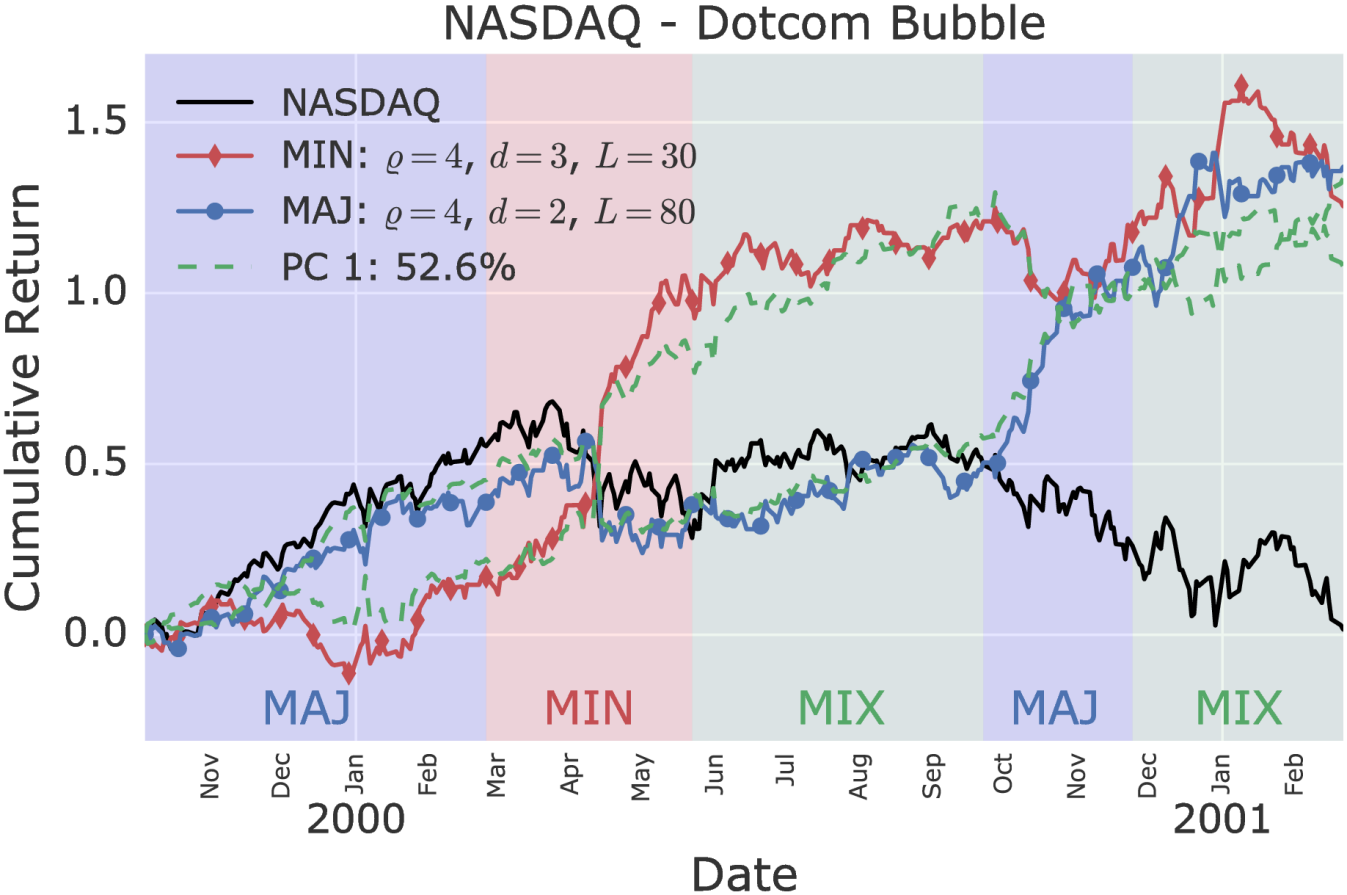
Most anomalous minority and majority model on the NASDAQ during the crash of the dotcom bubble. Six significant single agent models are found during the early peak and crash of the dotcom bubble. The first principal component (PC 1) explains 52.6% of the variance across these models. In particular, the first component is significantly shared by the minority and majority game models. Several successive regimes are distinguishable: (i) a majority regime (MAJ) building up to the peak; (ii) a strong minority regime (MIN) during the first crash in March 2000; (iii) a mixed regime in which the market remains flat; and finally (iv) a majority regime during the true burst of the bubble in September 2000.

Determining the first PC of the six anomalous models during the beginning of the dotcom bubble revealed that 52.6% of the variance is explained by the first PC over a 15 month time frame. The first PC is significantly shared between the minority and majority models at different delays, showing that persistent and anti-persistent predictable patterns are intertwined. Fig 3 shows the two most anomalous models, alongside with their projection onto the first principal component. The minority model is gaining momentum during the first half of the year 2000, in particular during the first crash in late March 2000. During the subsequent period, the synchronicity between the majority and minority models keeps maturating, until the bubble ruptures in October 2000 accompanied by a strong majority signal.

**Fig 4:**
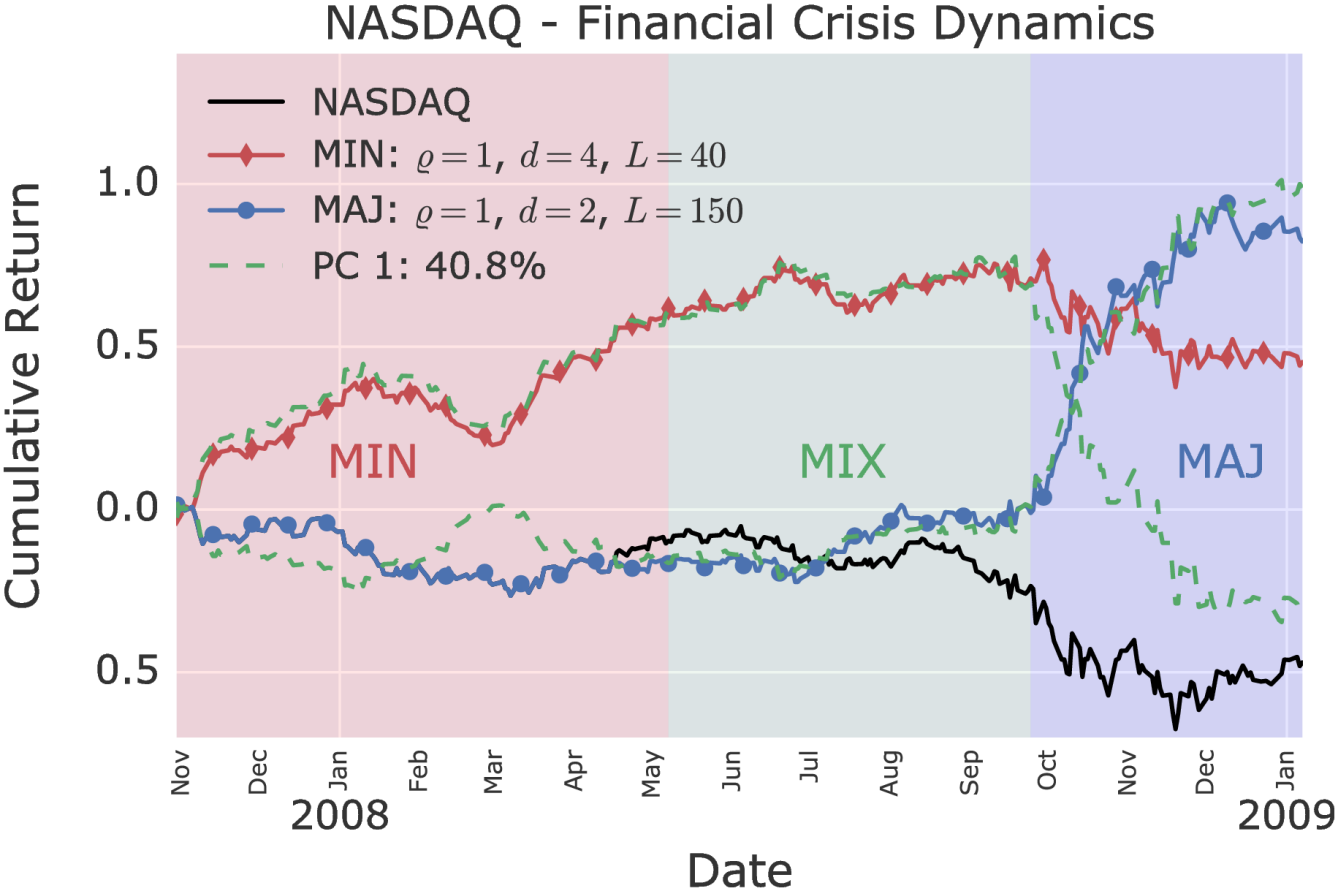
Most anomalous minority and majority model on the NASDAQ during the financial crisis. Eight significant single agent models are found during the financial crisis. The first principal component (PC 1) explains 40.8% of the variance across these models. In particular, the first component is significantly shared by the minority and majority game models. Three successive regimes are distinguishable: (i) an initial minority regime (MIN); (ii) a mixed regime (MIX) where the majority and minority models synchronize; and (iii) a strong majority regime during the burst of the bubble in September 2008.

As shown in Fig 4, the first PC of the eight anomalous models during the financial crisis explains 40.8% of the variance over a 15 month time frame. There as well, the minority and majority game models significantly share the first PC, but in more distinct time periods. The financial crisis visibly starts in late 2007 with a minority anomaly, while the majority model closely follows the buy-and-hold strategy. In April 2008, the minority and majority models start to synchronize, until a majority takes over in September 2008 and bursts the bubble.

The interplay between anomalous minority and majority patterns can be interpreted as the presence of two major groups of investors, confirming a study of individual traders that identified two investment styles [42]: contrarians profit takers; and positive feedback momentum traders.

### Heterogeneous two-agents models

The strong first principal component shared by the anomalously performing minority and majority models provides a novel angle to support the generally accepted hypothesis that the observed properties of financial markets result from the interplay between multiple agents with heterogeneous preferences [43–46]. The standard formulation of ABMs is to consider multiple agents, each possessing their specific preference (conditioning the formulation of the payoff and the game structure), a number of different strategies and time scales. Our present approach is more in the spirit of calibration, taken in the sense of designing ABMs that, when fed with real data, exhibit the best out-of-sample investment performance. The rational for this approach is that this success exemplifies a correct design of the principal ingredients that can match real markets. Rather than sampling random strategies from a large set, we thus combine two optimal single agent models selected from the previous section on single agent experiments, with different calibration lengths, pay-off structure, lags, and delays.

**Fig 5:**
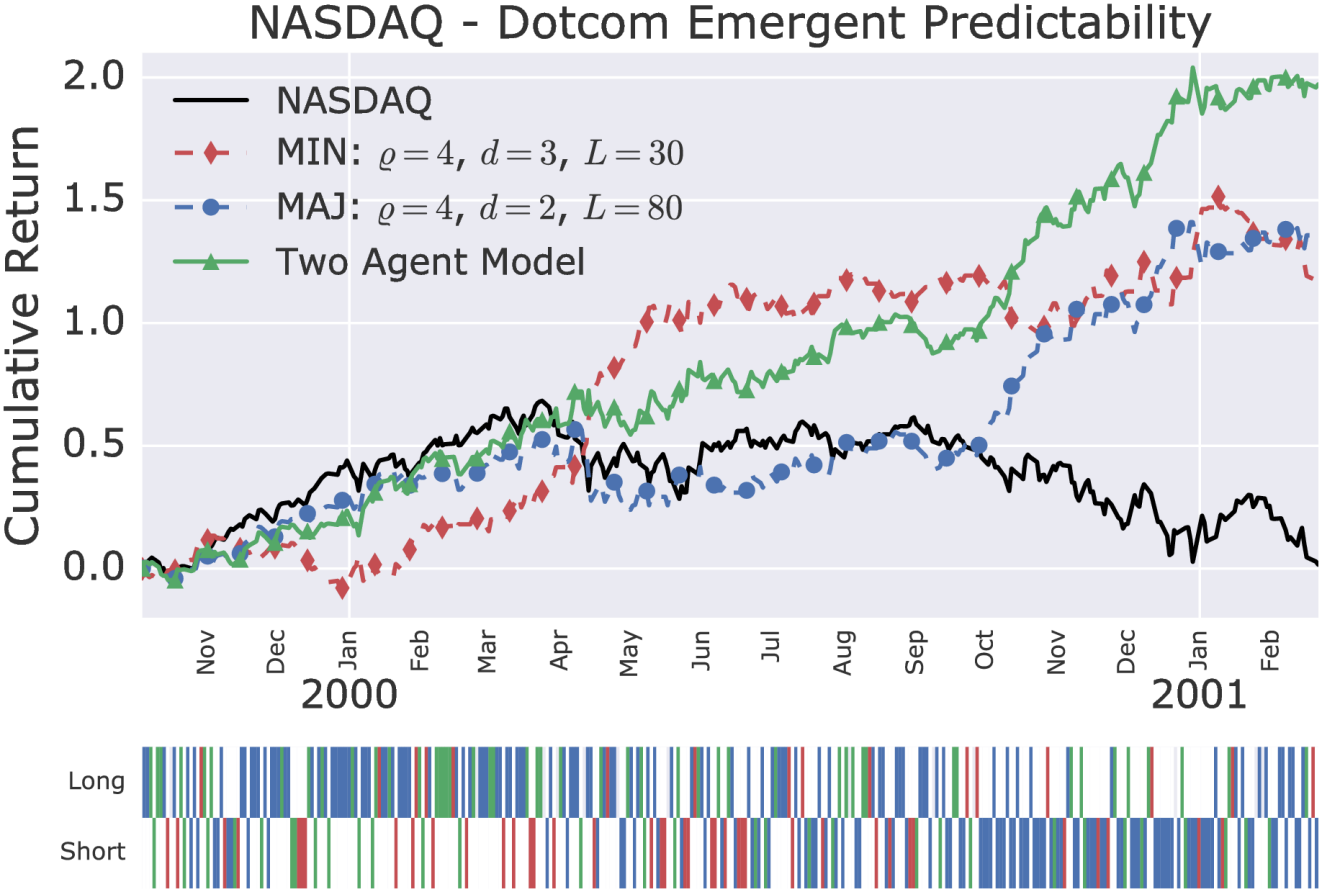
Two-agents model on the NASDAQ during the crash of the dotcom bubble. The two-agents model combines the forecast of the shown minority and majority agents as defined in (11). The lower panel indicates the long and short position taken by the two-agents model, including the following color coding: red, minority agent dominated; blue, majority agent dominated; green, both agents agreed.

**Fig 6:**
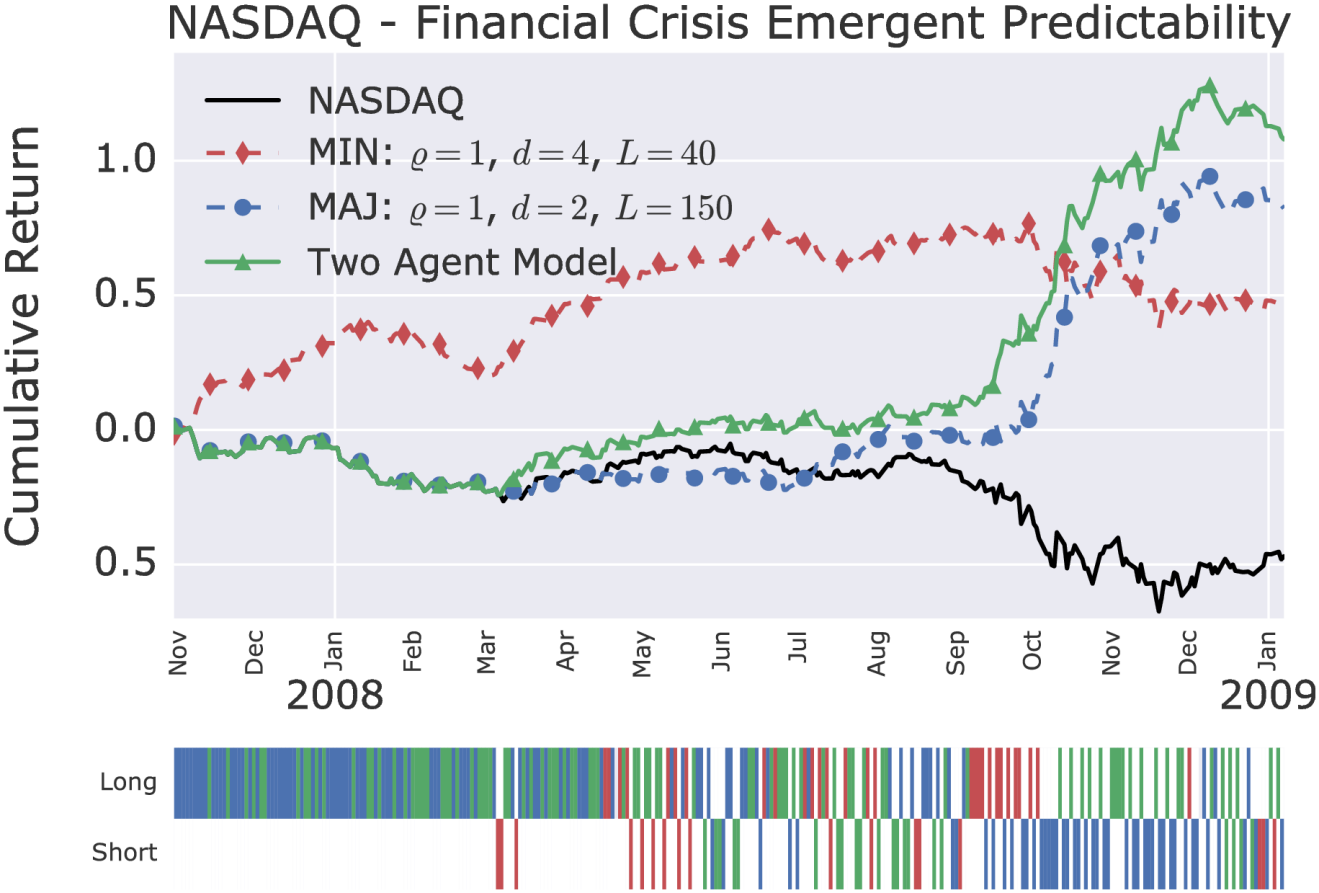
Two-agents model on the NASDAQ during the financial crisis. The two-agents model combines the forecast of the shown minority and majority models as defined in (11). The lower panel indicates the long an short position taken by the two agent model, with the following color coding: red, minority agent dominated; blue, majority agent dominated; green, both agents agreed.

The cumulative returns over time during the dotcom bubble and the financial crisis of the two-agents model, which combine the two best single agent models, are shown in Figs 5 and 6. Table 1 shows the improvement in daily Sharpe ratios of the two-agents model in comparison to the underlying single agent models. In both cases, the two-agents model out-performs above the 95% confidence level the single agent models.

**Table 1:** Sharpe ratio and buy-and-hold break-even transaction costs of the best single agent minority and majority model, and the two-agents model, during the dotcom bubble and financial crisis (F.C.). ‘Buy-and-hold break-even transaction costs’ is the fee level per one-way transaction (counted in bps, which stands for ‘basis points’, i.e. 0.01%) that would reduce the performance to that of the buy-and-hold strategy. The cumulative returns over time of the individual models are shown in Figs 5 and 6. The two-agents models outperform the single agent models in annualised Sharpe ratio above the 95% confidence level.

	Annualised Sharpe Ratio		Break-even cost (bps)	
Model	Dotcom	F. C.	Dotcom	F. C.
One Agent (MIN)	1.6	0.98	49	72
One Agent (MAJ)	1.9	1.7	65	137
Two-Agents Mix	2.6	2.3	93	138

## Conclusion

The out-performance of all single agent models by the constructed two-agent model is a non-trivial result, because there is no guarantee that the strength and timing of the anomalously high predictability in the individual models translates into an improved combined forecast. In a scenario where correct class probabilities are weak, and wrong class probabilities are strong, the individual anomalously high predictabilities could equally well cancel out when combined. We thus interpret these results as resulting from a procedure in which single agent models performed a kind of spectroscopy of the financial markets [47], identifying anomalous market dynamics that are precursors to an imminent rupture. Then, the combination of the two optimal individual agents into a two-agents model creates an emerging superior predictability, especially in the periods of bubbles and crashes. The out-performance of the two-agents model is thus strongly supporting the hypothesis that the observed market returns are the result of a dynamics emerging from the interplay between contrarian and momentum traders. While such a claim has been professed many times, the novelty is that our approach provides the first direct evidence in what is arguably the most important metric in this field, namely investment performance.

## Supporting information

S1 Appendix Proof of equivalence between agents and decision trees. (PDF)

S2 Appendix Directional accuracy. (PDF)

S3 Appendix Implementation. (PDF)

S1 Fig Anomalous time periods found in the S&P 500 with single agent models during the time period Jan. 1995 to Dec. 2015. A total of 2000 models are tested, resulting fromml: 2 games (MIN/MAJ) ×4 lags (*ϱ*) ×5 delays (*d*) ×50 calibration lengths (*L*). A total of 233 anomalous periods are found using a rolling window of one year, determining the models that exhibit statistically significant Sharpe ratio and directional accuracy after adjusting for multiple testing. The intensity of the color is determined by the maximum Sharpe ratio of the out-performing models. The black vertical lines are given as a visual aid for the timing of market anomalies.
(PDF)

S2 Fig Anomalous time periods found in the Dow Jones with single agent models during the time period Jan. 1995 to Dec. 2015. A total of 2000 models are tested, resulting fromml: 2 games (MIN/MAJ) ×4 lags (*ϱ*) ×5 delays (*d*) ×50 calibration lengths (*L*). A total of 297 Anomalous periods are found using a rolling window of one year, determining the models that exhibit statistically significant Sharpe ratio and directional accuracy after adjusting for multiple testing. The intensity of the color is determined by the maximum Sharpe ratio of the out-performing models. The black vertical lines are given as a visual aid for the timing of market anomalies.
(PDF)
