# Supplementary material for: Calibrating emergent phenomena in stock markets with agent based models
Source: PLoS One. 2018 Mar 2;13(3):e0193290. doi: 10.1371/journal.pone.0193290 (PMC5834198; doi:10.1371/journal.pone.0193290)
Supplement: S1 Appendix — (PDF) [file pone.0193290.s001.pdf]

Supporting Information

S1 Appendix. Proof of Equivalence Between Agents and Decision Trees

To find the strategy in $\mathbb{S}^{\varrho}$ with the highest performance, where the performance over one return history is defined in (5), the brute force approach is to compute the performance of every strategy individually over all possible histories. However, the computation of the performance (5) summed over all possible return histories can be optimized by rearranging the sums as

$$\langle U_{\pi,\,d,\,L}(s,\,t)\rangle \quad \propto \quad \sum_{\vec{r}\in\mathcal{R}^{\varrho}} \sum_{j\in\mathcal{L}|\vec{r}_j=\vec{r}} \pi\left(r_{j+d+1},\, s\left(\vec{r}_j\right)\right), \tag{1}$$

where $\mathcal{L} = \{t - \tilde{L} + d,\, \ldots,\, t-1-d\}$ and $\tilde{L} = L - \varrho$. Now the inner sum over a fixed history $\vec{r}$ can be maximized independently from the outer sum. Denoting by $p_{\vec{r},\,r}$ the probability of finding the transition $P\left(\vec{r}_t \to r_{t+d+1}\right)$ in the calibration window of length L, the performance function is given by

$$\langle U_{\pi,\,d,\,L}(s,\,t)\rangle \quad \propto \quad \sum_{\vec{r}\in\mathcal{R}^{\varrho}} \sum_{r\in\mathcal{R}} p_{\vec{r},\,r}\,\pi\left(r,\, s\left(\vec{r}\right)\right). \tag{2}$$

To maximize the inner sum, the best strategy is now entirely determined as

$$s\left(\vec{r}\right) = \underset{r'\in\mathcal{R}}{\operatorname{argmax}} \sum_{r\in\mathcal{R}} p_{\vec{r},\,r}\,\pi\left(r,\, r'\right). \tag{3}$$

When several values $r \in \mathcal{R}$ maximize the inner sum with a tigh, a random choice is made. The computation time for the probabilities $p_{\vec{r},\,r}$ is linear with respect to the size L of the calibration window, which makes the computation of the best strategy independent from the number of strategies in the space $\mathbb{S}^{\varrho}$.

Updating the best performing strategy from one time step to the next is achieved with a fixed computational cost. Removing the time step $t - \tilde{L} + d$ from $\mathcal{L}$, and adding the time step $t - d$, will affect at most two of the probabilities $p_{\vec{r},\,r}$, which are updated without recomputing the other probabilities. This change in the probabilities affects at most two of the inner sums in Equation (**??**), and therefore requires to update at most

two of the values of $s(\vec{r})$.

The decision tree predicts for each history (i.e. branch) the class with the highest probability. This prediction mechanism is equivalent to selecting the strategy with the highest performance for the majority game as defined in the Subsection on strategy performance in the main text. The probabilities $p_{\vec{r},r}$, used in Equation (**??**) to determine the strategy with the highest performance, are the class probabilities of the equivalent decision tree. Consequently, an agent endowed with the knowledge of all possible strategies, selecting at every time step the strategy with the highest performance on the past window of size L, is equivalent to an optimal decision tree calibrated on that window. In the case of the minority game, the tree prediction function is defined to return the class with the lowest probability.
