# Supplementary material for: Calibrating emergent phenomena in stock markets with agent based models
Source: PLoS One. 2018 Mar 2;13(3):e0193290. doi: 10.1371/journal.pone.0193290 (PMC5834198; doi:10.1371/journal.pone.0193290)
Supplement: S2 Appendix — (PDF) [file pone.0193290.s002.pdf]

Supporting Information

S2 Appendix. Directional Accuracy

The statistical significance of directional accuracy is given by a test of independence between the directions of the true and predicted returns. To test for independence, the samples consisting of a true and a predicted return direction are assigned to a 2×2 contingency matrix O. The row index corresponds to the direction of the true return, and the column index to the direction of the predicted return.

For contingency matrix O, with observation numbers $O_{i,\,j}$, the chi-squared is defined as

$$\hat{\chi}_d^2 = \sum_{i=1}^{2} \sum_{j=1}^{2} \frac{\left(O_{i,\,j} - \mathrm{E}_{i,\,j}\right)^2}{\mathrm{E}_{i,\,j}}, \tag{1}$$

where

$$\mathrm{E}_{i,\,j} = N \rho_{i\cdot} \rho_{\cdot j}, \quad N = \sum_{i=1,j=1}^{2,\,2} O_{i,\,j}, \tag{2}$$

$$\rho_{i\cdot} = \frac{1}{N} \sum_{j=1}^{2} O_{i,\,j}, \quad \text{and} \quad \rho_{\cdot j} = \frac{1}{N} \sum_{i=1}^{2} O_{i,\,j}. \tag{3}$$

The value $\hat{\chi}_d^2$ is used as the performance measure for directional accuracy. Under the null hypothesis of independence, the p-value of the estimated chi-square is

$$P\left(\chi_d^2 \geq \hat{\chi}_d^2\right) = \sqrt{\pi}\Gamma\left(\frac{1}{2}\right)^{-1} \mathrm{Erfc}\left(\sqrt{\frac{\hat{\chi}_d^2}{2}}\right). \tag{4}$$

The p-value is the probability of independent samples having a chi-squared value χ_d^2 larger than the observed value $\hat{\chi}_d^2$. This result shows that in the binary case the $\hat{\chi}_d^2$ value is normally distributed with mean zero.

The chi-squared value is better expressed in terms of the excess predictability

$$\Delta\rho_{i,\,j} = \frac{1}{N}\left(O_{i,\,j} - E_{i,\,j}\right), \tag{5}$$

which relates to the chi-squared as

$$\hat{\chi}_d^2 = \sum_{i=1}^{2} \sum_{j=1}^{2} \frac{2^2}{N} \left(N\Delta\rho_{i,\,j}\right)^2 = 16N\Delta\rho^2, \tag{6}$$

where $\Delta\rho$ is the mean excess predicatiblity, and $E_{i,\,j} = \frac{N}{2^2}$.
