# Supplementary material for: Calibrating emergent phenomena in stock markets with agent based models
Source: PLoS One. 2018 Mar 2;13(3):e0193290. doi: 10.1371/journal.pone.0193290 (PMC5834198; doi:10.1371/journal.pone.0193290)
Supplement: S3 Appendix — (PDF) [file pone.0193290.s003.pdf]

Supporting Information

S3 Appendix. Implementation

Single Agent

The single agent and two agent prediction models are implemented using the scikit-learn package in python. The multiple-testing adjusted p-values for the Sharpe ratio can be computed out of the box with the arch package in python. The implementation of the single agent model, as a wrapper of the DecisionTreeClassifier class, is provided below.

```
import numpy as np
from sklearn.tree import DecisionTreeClassifier



class OneAgent(object):
  def __init__(self, game, lags, delay, l):
    # Game is majority (MAJ) or
    # minority (MIN)
    self.game = game

    self.lags = lags
    self.delay = delay

    # Calibration window length L
    self.l = l

  def predict_proba(self, returns):
    # Local shortcuts
    p = self.lags
    d = self.delay
    l = self.l

    # Compute binary returns of
```

```
# up and down moves.
# Take only l (L) last returns
b_returns = [
  1 if r >= 0 else -1
  for r in returns[-l:]
]

# <- ϱ -><- d -><- output ->

# Compute inputs as an array
# with shape (L − ϱ − d, ϱ)
inputs = np.array([
  returns[i:-(p + d - i)]
  for i in range(p)
], dtype=float).transpose()

# Compute outputs matching to the inputs
outputs = b_returns[p+d:]

# Create a classification tree instance
tree = DecisionTreeClassifier(
  criterion="gini",
  splitter="best",
  max_depth=None,
  min_samples_split=2,
  min_samples_leaf=1,
  min_weight_fraction_leaf=0.,
  max_features=None,
  random_state=None,
  max_leaf_nodes=None,
  min_impurity_split=0,
  class_weight=None,
```

```
      presort=False
    )

    # Train the tree
    tree.fit(inputs, outputs)

    if d == 0:
      prediction_input = [b_returns[-p:]]
    else
      prediction_input = [b_returns[-(p+d):-d]]

    ps = tree.predict_proba(prediction_input)[0]

    # Threshold determination
    dp = ps[1] - ps[0] if len(ps) > 1 else ps[0]

    # Game determination
    return dp if self.game == "MAJ" else -dp

  def predict(self, returns):
    dp = self.predict_proba(returns)
    return np.sign(dp) if np.abs(dp) >= 0 else 0



def strategy_returns(returns, model):
  max_l = 500
  return [
    model.predict(returns[(i-max_l):i])
    for i in range(max_l, len(returns))
  ] * returns[max_l:]
```

Two Agents

The two agent model combines two single agents as implemented below.

```
import numpy as np



class TwoAgent(object):
  def __init__(self, agent1, agent2):
    self.agent1 = agent1
    self.agent2 = agent2

  def predict(self, returns):
    dp1 = self.agent1.predict_proba(returns)
    dp2 = self.agent2.predict_proba(returns)

    w1 = self.agent1.l * 2**self.agent2.lags
    w2 = self.agent2.l * 2**self.agent1.lags
    dp = (w1 * dp1 + w2 * dp2)/(w1 + w2)

    return np.sign(dp) if np.abs(dp) >= 0 else 0
```

Directional Accuracy

The p-value for the directional accuracy can be computed efficiently as an independence test on the confusion matrix, as implemented below.

```
from sklearn.metrics import confusion_matrix
from scipy.stats import chi2_contingency



def directional_p_value(true_returns, predicted_returns):
    # Ensure returns are binary sequences of up and down moves
    true_returns = [0 if r <= 0 else 1 for r in true_returns]
    predicted_returns = [0 if r <= 0 else 1 for r in predicted_returns]
```

```
# Compute confusion matrix
cm = confusion_matrix(true_returns, predicted_returns)

# Return p-value of Pearson independence test
return chi2_contingency(cm)[1]
```
