# Supplementary material for: Calibrating emergent phenomena in stock markets with agent based models
Source: PLoS One. 2018 Mar 2;13(3):e0193290. doi: 10.1371/journal.pone.0193290 (PMC5834198; doi:10.1371/journal.pone.0193290)
Supplement: S2 Fig — A total of 2000 models are tested, resulting fromml: 2 games (MIN/MAJ) ×4 lags (ϱ) ×5 delays (d) ×50 calibration lengths (L). A total of 297 Anomalous periods are found using a rolling window of one year, determining the models that exhibit statistically significant Sharpe ratio and directional accuracy after adjusting for multiple testing. The intensity of the color is determined by the maximum Sharpe ratio of the out-performing models. The black vertical lines are given as a visual aid for the timing of market anomalies. (PDF) [file pone.0193290.s005.pdf]

Supporting Information

S2 Figure. Anomalous Time Periods for the Dow Jones

The anomalous single agent models for the Dow Jones are shown in S2 Fig. The results are similar to the NASDAQ, strengthening the robustness of the methodology.

S2 Fig. Anomalous time periods found in the Dow Jones with single agent models during the time period Jan. 1995 to Dec. 2015. A total of 2000 models are tested, resulting from: 2 games (MIN/MAJ) $\times 4$ lags (ϱ) $\times 5$ delays (d) $\times 50$ calibration lengths (L). A total of 297 Anomalous periods are found using a rolling window of one year, determining the models that exhibit statistically significant Sharpe ratio and directional accuracy after adjusting for multiple testing. The intensity of the color is determined by the maximum Sharpe ratio of the out-performing models. The black vertical lines are given as a visual aid for the timing of market anomalies.
